# Nurses’ self-reported time estimation of anticoagulation therapy: a survey of warfarin management in long-term care

**DOI:** 10.1186/s12912-015-0058-x

**Published:** 2015-02-21

**Authors:** Aarti A Patel, Winnie W Nelson

**Affiliations:** Janssen Scientific Affairs, LLC, 1000 Route 202, Raritan, NJ 08869 USA

**Keywords:** Anticoagulants, Long-term care, Nursing shortage, Quality of care, Warfarin management, Monitoring

## Abstract

**Background:**

A nursing shortage in the United States has resulted in increased workloads, potentially affecting the quality of care. This situation is particularly concerning in long-term care (LTC) facilities, where residents are older, frailer, and may be receiving multiple medications for comorbidities, thus requiring a greater commitment of nurse time. We conducted a survey of LTC nurses to determine how much of their time each week is spent managing newly started and stable warfarin-treated residents.

**Methods:**

Forty LTC nurses validated the questionnaire to determine what protocols/procedures are involved in warfarin management. Twenty LTC nurses completed the survey, quantifying the time they spend on procedures related to warfarin management, and how often they performed each procedure for each resident each week.

**Results:**

The nurses reported that 26% of their residents were receiving warfarin; the majority (approximately 75%) of these residents began warfarin after admission to the facility. On average, the nurses spent 4.6 hours per week for treatment procedures and monitoring patients initiating warfarin therapy and 2.35 hours per week for each resident who was stable on warfarin therapy on admission. Overall, to care for an average number of newly initiated and stable warfarin patients in a medium-size LTC facility, staff nurses are estimated to spend 68 hours per week.

Study limitations include the potential for bias because of the small sample size, representativeness of the sample, and the possibility of inaccuracies in respondents’ self-reported time estimation of warfarin-related procedures.

**Conclusions:**

In the context of a well-documented and expanding nursing shortage in the United States, the substantial use of time and resources necessary to initiate, monitor, and manage warfarin treatment in elderly LTC patients is of concern. Until the problem of understaffing is resolved, implementation of therapies that are simpler and require less nursing time—e.g. the use of new oral anticoagulants in the place of warfarin—may be a way to free up nursing time for other essential care tasks.

**Electronic supplementary material:**

The online version of this article (doi:10.1186/s12912-015-0058-x) contains supplementary material, which is available to authorized users.

## Background

### The state of the nursing profession in the United States today

As more people live to older ages, there is an increasing need for healthcare services. Registered nurses (RNs) comprise the largest and most rapidly expanding segment of the healthcare workforce. According to the Bureau of Labor Statistics, unfilled positions for RNs are projected to reach 1.2 million by 2020 [1].

A shrinking economy appears to have eased the nursing shortage, but with an anticipated 32 million Americans gaining access to healthcare services as a result of the Patient Protection and Affordable Care Act, the need for nurses will become even more pressing and is projected to outstrip nurse availability [2,3].

### How the shortage of nurses affects patient care

A 2002 survey reported in the *New England Journal of Medicine* found that 53% of physicians and 65% of the public believe that a shortage of nurses increases the risk of medical errors [4]. Research on hospital-acquired infections showed that increasing a nurse’s load by merely one patient can result in higher rates of urinary tract and surgical-site infections [5]. Survey data from 40,000 RNs has also shown that, when linked with patient mortality data, increasing the number of patients per hospital nurse from four to eight results in a 1.26-fold greater risk of in-hospital mortality [6]. In a study that analyzed the records of approximately 198,000 patients across 43 care units, the mortality risk was up to 4% higher in units that were below target staffing levels than in fully staffed units [7].

With a growing elderly population, the shortage of nurses is expected to be particularly acute in long-term care (LTC) facilities. Elderly LTC residents require close attention and may have multiple conditions (e.g. diabetes, heart failure, hypertension, gastrointestinal or urologic disorders, and chronic pain) for which they receive numerous chronic medications, many of which must be administered multiple times throughout the day, imposing considerable demands on nurses’ time.

### Warfarin in long-term care

The anticoagulant warfarin is a prime example of a challenging and difficult-to-manage drug that is commonly used in the LTC setting. Warfarin is generally used to treat residents with procoagulant and cardiovascular conditions, including nonvalvular atrial fibrillation, the prevalence of which is reported to range from 7.5–17% among LTC residents [8-11]. Although efficacious as an anticoagulant, patients on warfarin require close monitoring to maintain optimal coagulation levels: if under-coagulated, the patient is at risk of a thromboembolic event; if over-coagulated, there is a risk of bleeding complications. In addition, warfarin has a narrow therapeutic window and interacts with several common drugs (e.g. lipid-lowering agents, antihypertensives, gastrointestinal drugs, analgesics, and anti-infectives) as well as with food and alcohol. Therefore, patients treated with warfarin require regular monitoring of international normalized ratio (INR) values to ensure therapeutic efficacy and avoid potentially life-threatening adverse events. In the LTC setting, frequent INR monitoring as well as other patient factors, such as advanced age, comorbid conditions, and polypharmacy, make the management of warfarin particularly time consuming.

There is limited evidence in the literature examining the amount of time RNs and licensed practical nurses (LPNs) spend managing LTC residents newly initiated or stable on warfarin therapy. With healthcare resources scarce, analysis of the time involved in LTC patient management can inform decisions on the allocation of healthcare resources to ensure that they are efficiently utilized.

We undertook a primary semi-quantitative web-based survey of LTC nurses who were responsible for the care of LTC residents who received warfarin. Our objectives were to determine the specific nursing tasks related to warfarin therapy and estimate the time requirements for warfarin management, treatment initiation, and monitoring.

## Methods

Before constructing our survey, we conducted a literature search of peer-reviewed articles specific to warfarin management to develop a comprehensive list of activities performed by nurses during the course of managing new and stable warfarin residents in LTC facilities. We validated the accuracy of our findings by surveying a group of registered or licensed LTC nurses who were selected from a proprietary database including nurses throughout the United States. Nurses were contacted directly by e-mail, and respondents were screened to ensure that licensure requirements were met. Nurses who responded to the survey helped to refine the list of tasks and were also given the opportunity to propose adding or deleting tasks from the list. Based on nurses’ feedback, we created a follow-up survey (Additional file 1), in which 20 nurses (15 RNs, five LPNs) in LTC facilities were asked to (1) confirm these steps and procedures with respect to their individual responsibilities, and (2) provide an estimate of total completion times for each task, and report how often they perform each task each week for each resident receiving warfarin. For residents newly initiated on warfarin therapy, survey respondents were asked to report three categories of tasks: initiation (activities or tasks to start patients on warfarin), monitoring (INR testing and monitoring for bruising), and management (activities or tasks related to monitoring documentation, patient education, and physician/pharmacist consultation). For stable warfarin residents, only monitoring and management had to be reported. Participants were required to spend at least 50% of their daily time in direct patient care, per their assessment, and to have previous experience managing warfarin treatment. Respondents were asked to only estimate and self-report time that was actively spent documenting/performing warfarin-related procedures or educating residents (e.g. dietary consultations to discuss any potential drug-food interactions, medication profiles to account for potential drug-drug interactions, health education, and the need for ongoing monitoring). This time was self-reported and estimated. Rather than report the number of beds in their facilities, this was reported as a range: small (1–99 beds), medium (100–199 beds), and large (≥200 beds). To minimize geographical bias, only two nurses per state were permitted to participate in the survey.

On the basis of the estimates provided by the LTC nurses participating in the survey, the average time estimated per task is calculated as the number of times the task is performed per resident multiplied by the amount of time the task takes to complete. The total estimated time in residents newly initiated on warfarin therapy and stable warfarin residents is then derived by summing the average time per task over all the tasks per week. The total number of nursing hours was estimated based on the average percentage of warfarin residents (as a fraction of all residents in the facility) assuming a medium-size (100–199 beds) LTC facility. Because the percentage of newly initiated and stable warfarin patients was not reported, our analysis assumes that the majority of residents in the LTC facility were stable warfarin patients, providing for a more conservative estimate. The average time nurses estimated per week for newly initiated or stable warfarin residents within a medium-size facility is calculated as the number of warfarin residents multiplied by the amount of time per warfarin resident. The total number of hours LTC nurses spent managing residents receiving warfarin is derived by summing the total number of hours for both newly initiated and stable warfarin residents.

## Results

Forty qualified respondents validated and revised the list of procedures and other steps incorporated in the quantitative survey (30 RNs, 10 LPNs), while 20 were involved in the confirmation of these tasks and follow-up time-estimation survey (15 RNs, five LPNs). Because two surveys were sent, the respondents who participated in the first survey were not the same as those who participated in the second. However, all survey respondents took part in the validation and confirmation of the tasks included in the second questionnaire (N = 40). All respondents were nurses in LTC facilities and spent at least 50% of their time each day in direct patient care. They worked in a mix of nursing care facilities: skilled nursing facility (n = 39; inpatient post-acute care [12]); intermediate-care facility (n = 4; provides level of nursing care higher than the general ward without intensive care [13]), and a subacute facility (n = 1; inpatient intensive rehabilitative medical care between skilled nursing and acute hospital care [14]). (Some of the 40 institutions provided mixed services and were counted twice.) Of these facilities, 38% were categorized as small (1–99 beds), 43% as medium (100–199 beds), and 20% as large (≥200 beds). The majority of LTC facilities (87.5%) were owned and operated by the state, county, or local government.

Respondents estimated from their experience that 26% of the residents in their units of their LTC facilities were receiving warfarin. Of these patients, one-quarter were already receiving warfarin upon admission to the facility; three-quarters were initiated on warfarin therapy during their stay.

### Protocols and procedures for newly initiated warfarin residents

Median completion time per patient for procedures related to warfarin initiation is shown in Table 1. Considerable time is spent on dietary consultations to discuss any potential drug-food interactions, medication profiles to account for potential drug–drug interactions, and patient education regarding these concerns and the need for ongoing monitoring of anticoagulation levels. The median time spent on protocols specific to monitoring newly initiated residents is shown in Table 2.

**Table 1:** **Median completion time spent on warfarin treatment initiation protocols/procedures**

**Procedure**	**Completion time per patient (minutes)**
Baseline INR	20
Baseline PT and CBC	35
Dietary consult	20
Medication profile	20
Patient education	20
CBC at initiation of warfarin therapy	15
PRN order for INR levels when complications are suspected	15

CBC = complete blood count; INR = international normalized ratio; PRN = *pro re nata* (as needed); PT = prothrombin time.

**Table 2:** **Median time spent on protocols/procedures related to monitoring residents newly initiated on warfarin therapy**

**Protocols/procedures**	**Completion time per patient (minutes)**	**Weekly frequency**
PT/INR until the warfarin dose is established	15	2
Document/monitor laboratory values on the anticoagulation care/flow sheet	5	2
Monitor for bruising, bleeding, symptoms of gastrointestinal bleeding	5	7
Routine physical assessment to monitor for signs and symptoms of bleeding	8	7

INR = international normalized ratio; PT = prothrombin time.

### Protocols and procedures for stable warfarin residents

Median time spent per patient for procedures related to monitoring stable warfarin residents is shown in Table 3. On average, each task takes nurses between 5 and 10 minutes, with monitoring and documenting laboratory values requiring the most time. Thus, LTC nurses spend just over 1 hour per week performing the tasks required for monitoring anticoagulation levels in residents who are stable on warfarin therapy.

**Table 3:** **Median time spent on protocols/procedures related to monitoring residents who are stable on warfarin therapy**

**Protocols/procedures**	**Completion time per patient (minutes)**	**Weekly frequency**
Document/monitor laboratory values on the anticoagulation care/flow sheet	10	1
INR	5	1
Monitor for bruising/bleeding, symptoms of gastrointestinal bleeding	5	7
Routine physical assessment to monitor for signs/symptoms of bleeding	5	3

INR = international normalized ratio.

### Non-monitoring management tasks for all warfarin residents

All residents receiving warfarin require an investment of time that is not related to monitoring, as shown in Table 4. Each of these management tasks takes relatively little time—between 5 and 12 minutes—with documentation requiring the most time. Nevertheless, in the aggregate, these tasks require an average of 1.25 hours per week per warfarin resident.

**Table 4:** **Median time spent on protocols/procedures not related to monitoring residents who are stable on warfarin therapy**

**Protocols/procedures**	**Completion time per patient (minutes)**	**Weekly frequency**
Document therapy/bleeding precautions on the patient’s care plan	12	1
Order next INR test	5	1
Consult referring physician for all coagulation test results out of range	5	1
Adjust the warfarin dose when the therapeutic goals are not being met	5	1
Document dosing changes in the patient’s chart	10	1
Manage drug-related problems with the physician	5	1
Establish patient’s scheduled dosing time	5	1
Provide routine education	5	3
Make CNAs aware of warfarin residents and care plan	5	3

CNA = Certified Nursing Assistant; INR = international normalized ratio.

### Estimated nursing time per week for newly initiated and stable warfarin residents

Respondents reported spending 2.35 hours per week for each stable resident and 5.9 hours per week for each newly initiated warfarin patient. For stable patients, the time spent included 1.05 nursing hours per week for monitoring and 1.3 nursing hours per week for managing. For newly initiated patients, 2.4 nursing hours per week were required for warfarin initiation, 2.2 hours per week for monitoring, and 1.3 hours per week for management.

Based on a hypothetical medium-sized LTC facility (100 beds), when both newly initiated (n = 2) and stable warfarin residents (n = 24) were taken into account, including all the time devoted to warfarin initiation, monitoring, and management tasks, LTC nurses reported an average of 68 hours per week required for warfarin-related tasks, 11.8 hours per week for newly initiated, and 56.4 hours per week for residents already stable on warfarin therapy.

### Underestimation of nursing time

Nurses were asked to subjectively estimate time spent managing a single warfarin patient and all of their warfarin patients. This subjective assessment was asked before the quantitative survey questions that determined the itemized time spent on warfarin management, to reduce potential bias. Of all respondents, 43% estimated that they spent up to 30 minutes per week managing a single warfarin resident; only 10% believed that they spent 46–60 minutes per week. Approximately half of respondents (48%) estimated that they spent more than 3 hours per week managing all of their warfarin-treated residents; the remaining 52% believed that they spent 3 hours or less.

## Discussion

To our knowledge, this study is the first to assess the time taken for nurses to monitor and manage warfarin therapy in warfarin patients in an LTC setting. Based on the self-time estimation responses of 15 RNs and five LPNs, our study showed that nurses in LTC facilities in the United States spent an average of 2.4 hours per week (2.4 hours per patient) performing warfarin initiation procedures, 1.05–2.4 hours per week (25–28 minutes per patient) performing warfarin monitoring tasks, and 1.3 hours per week (57 minutes per patient) performing procedures related to warfarin management. A survey-based study by Andersson and colleagues showed similar findings with regard to the time taken by nurses to perform warfarin monitoring procedures in a primary healthcare setting in Sweden [15]. Their study reported an average of 21.4 minutes per patient to perform INR monitoring procedures (preparation, direct patient contact, and follow-up). Although the time taken for nurses to perform warfarin monitoring procedures are similar between the two studies, the study by Andersson only estimated the time taken to perform INR monitoring procedures; it did not take into account the time spent for initiation and management procedures. Moreover, the study was conducted in a primary healthcare setting and most likely included a more diverse patient demographic, including younger and potentially healthier individuals. Thus, while the results of our study may not be entirely translatable, there is previous work that utilizes self-reported estimates or modeled estimates of resource utilization to determine the time required to complete specific tasks [16].

Although the LTC setting would seem to be ideal for warfarin therapy, because laboratory monitoring, compliance, dose adjustment, and drug-drug interactions can be controlled for, our study found that the estimated time taken by nurses to perform procedures involved in such management requires substantial resources (up to 68 nursing hours per week when both newly initiated and stable warfarin patients are taken into account). This finding is in stark contrast to the subjective assessment provided by the nurses, who reported an average of 3 hours each week to manage all of their warfarin residents. This discrepancy in time utilization is likely due to the repetitive and routine nature of the procedures, which may result in a tendency to underestimate the time taken to complete each task. Additionally, when asked to estimate the time taken to perform these procedures, respondents may have based their responses on the time taken to monitor and manage stable warfarin residents, which may have considerably reduced the subjective time estimate, as initiation procedures were by far the most time-consuming based on the quantitative time estimates. It is also possible that the reported time could be inaccurate if nurses estimated separate amounts of time for discrete tasks that were consolidated and performed at the same time, or conversely, if they suddenly needed to tend to another patient and reported the time altogether as one warfarin-related task. Furthermore, nursing leaders responsible for time allocation may overlook opportunities to improve warfarin management processes if actual procedure times are unintentionally minimized.

This study was subject to several limitations, including the potential for bias due to a small sample size, representativeness of the sample, and the possibility of inaccuracies in self-time estimation by respondents because of human error or overlap of warfarin-related tasks with routine physical assessments. Because our study methods were predicated on a market-based research model using a survey design, details on recruitment, respondent demographic characteristics (e.g. years of experience), or institution (e.g. number of beds) were not collected. Based on our calculations, the use of a hypothetical medium-sized LTC facility (100–199 beds) may have led to potential over- or under-estimation of the weekly and total time taken by nurses to perform warfarin-related procedures. In addition, our study was not powered to determine differences in the times taken to perform warfarin-related procedures based on nurse designations and type of LTC. Because the two nurse populations surveyed were different (15 RNs, five LPNs), a statistical analysis of the differences in time estimations may not have been optimal. Because our study only captured the time spent by nurses monitoring and managing warfarin patients, additional work that was carried out by other types of personnel (e.g. laboratory staff and assistant nurses) and other factors not related to time estimation (e.g. quality of care) have not been accounted for. Furthermore, there is a potential double-counting of facilities that provide more than one of the services outlined in the types of facilities that were considered in this study. Therefore, the results are to be regarded as a conservative estimate of the time resources required for carrying out warfarin-related procedures in LTC facilities in the United States. Additional prospective studies are needed to examine differences in the time taken to perform warfarin-related procedures based on nurse designation and type of LTC.

## Conclusions

In the context of a well-documented and expanding nursing shortage in the United States, the substantial use of time and resources necessary to initiate, monitor, and manage warfarin treatment in elderly LTC patients is of concern. As a result of insufficient numbers of nurses being trained to care for a growing population of older patients, even (or perhaps especially) the most committed nurses are becoming frustrated, demoralized, and burned out, potentially further reducing the ranks of qualified professionals and posing an increased threat to optimal anticoagulation management [17,18]. This is particularly worrisome in the LTC setting, where the growing disparity between the increasing number of elderly individuals and the decreasing number of those who are charged with caring for them threatens to put frail and ill individuals at risk. Until the problem of understaffing is resolved, it would seem reasonable to maximize nurse efficiencies by making it possible for fewer nurses to provide optimal care. One solution, among others, might be to replace therapies that place a substantial burden on nurses with more efficient care models. Newer oral anticoagulants are available that have been shown to be safe and effective [19-21], as well as cost-effective, in older patients [22]. These agents have stable pharmacokinetics and fixed dosing. As a result, although regular assessments to monitor for symptoms of gastrointestinal or other bleeding are still necessary with the newer, target-specific oral anticoagulants, the routine INR monitoring, documentation of laboratory test results, dietary education, and dosing changes that are required with warfarin treatment are no longer needed with the newer agents. Adopting these newer anticoagulants could free up LTC nurses for other pressing patient responsibilities.

## Additional file

Additional file 1:
**Steps and procedures related to warfarin management in long-term care.**

## Abbreviations

CBC: Complete blood count
CNA: Certified nursing assistant
LPN: Licensed practical nurse
INR: International normalized ratio
LTC: Long-term care
PRN: *pro re nata* (as needed)
PT: Prothrombin time
RN: Registered nurse
