# Supplementary material for: Nurses’ self-reported time estimation of anticoagulation therapy: a survey of warfarin management in long-term care
Source: BMC Nurs. 2015 Feb 21;14:8. doi: 10.1186/s12912-015-0058-x (PMC4336714; doi:10.1186/s12912-015-0058-x)
Supplement: Additional file 1: — Steps and procedures related to warfarin management in long-term care. [file 12912_2015_58_MOESM1_ESM.doc]

**Additional file 1 Steps and procedures related to warfarin management in long-term care**

**Section 1: Treatment initiation**

| **Protocol/procedures related to newly initiated warfarin resident management and monitoring** | **How many minutes per task/per patient?** |
| --- | --- |
| Order and obtain a baseline INR on all newly admitted residents to determine current levels |  |
| Order and obtain baseline PT and CBC prior to the initiation of therapy |  |
| Order a dietary consult to assess the patient’s vitamin K intake |  |
| Assess the patient’s medication profile for drug-drug interactions that may impact the effects of warfarin |  |
| Provide patient education (e.g., the therapeutic effects of therapy, monitoring, dietary restrictions, and drug interactions) |  |
| Obtain a CBC at initiation of warfarin therapy |  |
| Obtain a PRN order for stat INR levels any time complications are suspected |  |

**Section 2:** Monitoring

| **Protocol/procedures related to warfarin resident monitoring** | **How many minutes per task/per new patient?** | **How many minutes per task/per stable patient?** | **How often was each task performed each week?** | **How many patients per task in a week?** |
| --- | --- | --- | --- | --- |
| Obtain a daily PT/INR until the warfarin dosage is stabilized |  |  |  |  |
| Monitor anticoagulant care/flow sheet |  |  |  |  |
| Measure INR for stable patients |  |  |  |  |
| Monitor every shift for bruising, bleeding, symptoms of gastrointestinal bleeding |  |  |  |  |
| Routine physical assessment to monitor for signs and symptoms of bleeding |  |  |  |  |

**Section 3:** Management

| **Protocol/procedures related to warfarin resident management** | **How many minutes per task/per patient?** | **How many patients per week for each task?** |
| --- | --- | --- |
| Use a standardized form for warfarin orders (e.g., Daily Warfarin Flow Sheet) |  |  |
| Document anticoagulant therapy and bleeding precautions on the patient’s care plan |  |  |
| Order and assign a date/time for the next INR test |  |  |
| Consult the referring physician for all coagulation test results significantly out of range per protocol |  |  |
| Adjust the anticoagulant dose as needed when the therapeutic goals of treatment are not being met |  |  |
| Document dosing changes in the patient chart/flow sheet |  |  |
| Assess, manage, document, and communicate drug-related problems (except for minor problems) to the physician |  |  |
| Make CNAs aware of which residents are receiving Coumadin and the special care they may need |  |  |
| Routine education regarding safety measures to be exercised while on warfarin therapy |  |  |
| Standardize dosing |  |  |
| Pharmacist also evaluates residents for drug-drug interactions |  |  |

CBC = complete blood count; CNA, certified nursing assistant; INR = international normalized ratio; PRN = *pro re nata* (as needed); PT = prothrombin time.
